# Effect of Ozone on Intestinal Epithelial Homeostasis in a Rat Model

**DOI:** 10.5041/RMMJ.10181

**Published:** 2015-01-29

**Authors:** Igor Sukhotnik, Alona Starikov, Arnold G. Coran, Yulia Pollak, Rima Sohotnik, Ron Shaoul

**Affiliations:** 1 Laboratory of Intestinal Adaptation and Recovery, The Bruce Rappaport Faculty of Medicine, Technion-Israel Institute of Technology, Haifa, Israel;; 2 Department of Pediatric Surgery, Bnai Zion Medical Center, Haifa, Israel;; 3 Section of Pediatric Surgery, C.S. Mott Children’s Hospital and University of Michigan Medical School, Ann Arbor, MI, USA; 4 Division of Pediatric Gastroenterology and Nutrition, Meyer Children’s Hospital of Haifa, Rambam Medical Center, Haifa, Israel

**Keywords:** Apoptosis, enterocyte, intestine, ozone, proliferation

## Abstract

**Background:**

The positive effects of ozone therapy have been described in many gastrointestinal disorders. The mechanisms of this positive effect of ozone therapy are poorly understood. The purpose of the present study was to investigate whether the use of ozone may potentiate the gut intestinal mucosal homeostasis in a rat model.

**Methods:**

Adult rats weighing 250–280 g were randomly assigned to one of three experimental groups of 8 rats each: 1) Control rats were given 2 mL of water by gavage and intraperitoneally (IP) for 5 days; 2) O_3_-PO rats were treated with 2 mL of ozone/oxygen mixture by gavage and 2 mL of water IP for 5 days; 3) O_3_-IP rats were treated with 2 mL of water by gavage and 2 mL of ozone/oxygen mixture IP for 5 days. Rats were sacrificed on day 6. Bowel and mucosal weight, mucosal DNA and protein, villus height and crypt depth, and cell proliferation and apoptosis were evaluated following sacrifice.

**Results:**

The group of O_3_-IP rats demonstrated a greater jejunal and ileal villus height and crypt depth, a greater enterocyte proliferation index in jejunum, and lower enterocyte apoptosis in ileum compared to control animals. Oral administration of the ozone/oxygen mixture resulted in a less significant effect on cell turnover.

**Conclusions:**

Treatment with an ozone/oxygen mixture stimulates intestinal cell turnover in a rat model. Intraperitoneal administration of ozone resulted in a more significant intestinal trophic effect than oral administration.

## INTRODUCTION

Ozone (O_3_) is found in nature in the form of a gas consisting of three atoms of oxygen in a cyclic structure, and is continually produced from and converted into molecular O_2_. Medical ozone therapy uses a mixture of ozone and oxygen and appears to be a safe, economical, effective treatment for patients with advanced ischemic conditions, peritonitis, infected wounds, chronic skin ulcers, initial gangrene, burns, and other maladies.^1^–^3^ The mechanisms of the positive effects of ozone are not yet fully understood and are mainly attributed to improvement in blood circulation and oxygen delivery to damaged tissues, enhanced general metabolism, up-regulation of cellular antioxidant enzyme activity, activation of the immune and neuro-endocrine systems, and enhancement in the release of growth factors from platelets.^4^,^5^ The ozone/oxygen mixture has a modulator effect on the phagocytic activity of peritoneal and alveolar macrophages.^5^,^6^ It has been shown that O_3_ increases the activity of antioxidant enzymes, such as glutathione peroxidase and catalase. These enzymes protect the host against pathophysiological conditions mediated by reactive oxygen species (ROS).^2^,^4^ Ozonized autohemotherapy is specifically suited for the treatment of vascular diseases such as stroke, peripheral vascular disease, as well as chronic heart disease. It can also be useful for supportive therapy in chronic infectious diseases, diabetes, and cancer, but it is not considered as curative in these conditions. On the other hand, cutaneous and ulcerative infections are well treated with topical application of ozonated oils and ozonated water. Such derivatives have also been used in dentistry (primary carious lesions). Finally, a gaseous oxygen/ozone mixture is very effective in the treatment of herniated discs.^6^

The positive effects of ozone therapy have been described in many gastrointestinal disorders. Ozone preconditioning decreased the tissue damage and increased the antioxidant enzyme activity in an experimental model of methotrexate-induced intestinal injury.^7^ The use of ozone reduced the incidence of postoperative inflammatory complications, protected intestinal anastomosis, and reduced mortality in an animal experiment.^8^ Ozone therapy decreased inflammation, edema, and oxidative stress in experimental colitis in a rat model.^9^ The mechanisms of this positive effect of ozone therapy in the gastrointestinal tract are poorly understood.

The purpose of the present study was to evaluate the effect of ozone therapy on intestinal mucosal homeostasis in rats.

## MATERIALS AND METHODS

The study was approved by the Technion–Faculty of Medicine Ethics Committee for Animal Studies, Haifa, Israel. Male Sprague–Dawley rats weighing 250–280 g were randomly assigned to one of three experimental groups of 8 rats each: 1) Control rats (Group A) were given 2 mL of water orally by gavage and intraperitoneally (IP) for 5 days; 2) A group of O_3_-PO rats were treated with 2 mL of ozone/oxygen mixture orally by gavage and with 2 mL of water intraperitoneally for 5 days; 3) A group of O_3_-IP rats were treated with 2 mL of water orally by gavage and 2 mL of an ozone/oxygen mixture intraperitoneally for 5 days. On the morning of the sixth day, animals were anesthetized with intraperitoneal sodium pentobarbital (45 mg/kg) and were then sacrificed by inducing an open pneumothorax. The small intestine was removed and divided into two segments: proximal jejunum and terminal ileum. Each segment was washed with ice-cold saline, weighed, and the weight per cm of bowel length was calculated. The segments were opened, bowel circumference was measured in three different sites, and mean bowel circumference was calculated. Mucosa was scraped off and weighed. DNA and protein were extracted using TRIzol reagent as described by Chomczynski.^10^ Final concentrations of DNA and protein were quantified by spectrophotometry, and their content in intestinal mucosa was calculated as µg/cm bowel length/100 g body weight.

### Histological Changes

The intestine was opened on the antimesenteric border and washed with iced saline. Pieces of proximal jejunum immediately distal to the ligament of Treitz and at the terminal ileum were fixed for 24 hours in 5% formalin, washed with absolute alcohol, and embedded in paraffin. Transverse 5 μm sections were prepared in a standardized fashion and were stained with hematoxylin and eosin. The study was performed blindly. Ten villi and crypts were selected for the microscopic analysis, using a 10×4 magnifying lens. Histological images were loaded on a 760×570 pixel resolution buffer using a computerized image analysis system composed of a trichip RGB video-camera (Sony, Tokyo, Japan), installed on a light microscope (Zeiss, Munich, Germany) and attached to an IBM compatible personal computer (Pentium III, MMX, 450 MHz, 125 MB RAM), equipped with a frame grabber. Images were captured, digitized, and displayed on a high-resolution color 17-inch monitor. The villus height and crypt depth were measured using the Image Pro Plus 4 image analysis software (Media Cybernetics, Baltimore, MD, USA) and were expressed in µm.

### Enterocyte Proliferation and Apoptosis

Crypt cell proliferation was assessed using 5-bromodeoxyuridine (5-BrdU). Standard BrdU labeling reagent (Zymed Laboratories, Inc., San Francisco, CA, USA) was injected intraperitoneally at a concentration of 1 mL/100 g body weight 2 hours before sacrifice. After paraffin removal, rehydration, and peroxidase inhibition, sections (5 μm) were successively incubated with a biotinylated monoclonal anti-BrdU antibody system provided in a kit form (Zymed Laboratories, Inc., San Francisco, CA, USA). An index of proliferation was determined as the ratio of crypt cells staining positively for BrdU per 10 crypts.

Immunohistochemistry for caspase-3 (caspase-3 cleaved concentrated polyclonal antibody; dilution 1:100; Biocare Medical, Walnut Creek, CA, USA) was performed to identify apoptotic cells using a combination of the streptovidin–biotin–peroxidase method and microwave antigen retrieval on formalin-fixed, paraffin-embedded tissues according to the manufacturer’s protocols. For each group, the number of stained cells was counted in one section separately in villi and crypts in areas without necrosis. The apoptotic index (AI) was defined as the number of apoptotic cells per 10 villi and the number of apoptotic cells per 100 crypts. All measurements were performed by a qualified pathologist who was blinded as to the source of intestinal tissue.

### Statistical Analysis

All data are presented as the mean±SD. Differences between experimental groups were tested for statistical significance (*P*<0.05) using the non-parametric Kruskal–Wallis ANOVA test, followed by the corrected Mann–Whitney test.

## RESULTS

### Intestinal Mucosal Parameters

**Figure 1.**
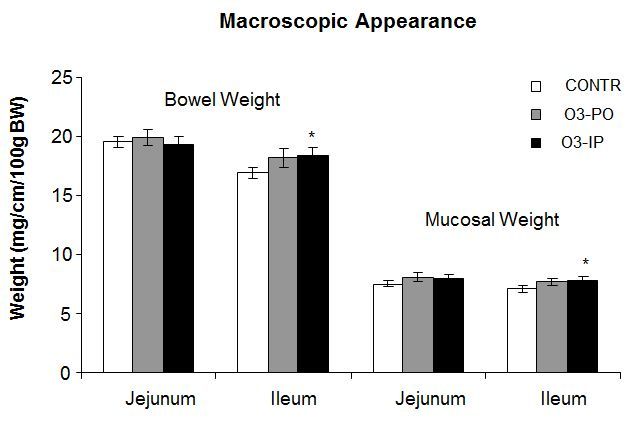
**Effect of Ozone on Bowel and Mucosal Weight.** Total bowel and mucosal weight for each segment is given as mg/cm of intestinal length per 100 g body weight. Values are provided as mean±SE. IP, intraperitoneal; O_3_, ozone; PO, per os (oral route administration). ^*^P<0.05 versus control.

**Figure 2.**
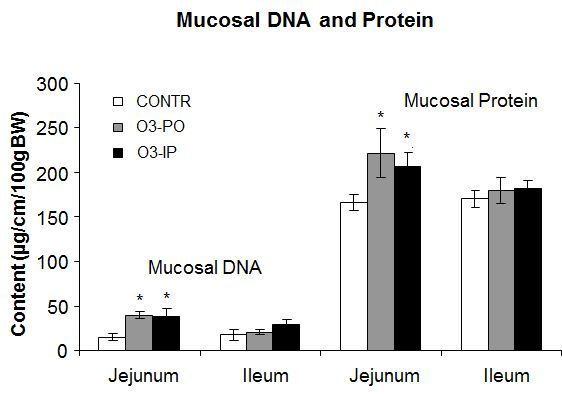
**Effect of Ozone on Mucosal DNA and Protein.** DNA and protein concentrations in intestinal mucosa were calculated as µg/cm bowel length per 100 g body weight. Values are provided as mean±SE. IP, intraperitoneal; O_3_, ozone; PO, per os (oral route administration). ^*^P<0.05 versus control.

The group of O_3_-PO rats (Group B) showed a trend toward an increase in bowel weight in ileum and mucosal weight in jejunum; however, this trend was not statistically significant (Figure 1). The O_3_-IP rats (Group C) demonstrated a significant increase in bowel (18.4±0.7 versus 17±0.4 mg/cm/100 g, *P*<0.05) and mucosal (7.9±0.3 versus 7.1±0.3 mg/cm/100 g, *P*<0.05) weight in ileum compared to control animals (Group A). Oral administration of ozone (Group B) resulted in a significant increase in mucosal DNA (2-fold increase, *P*<0.001) and protein (33% increase, *P*<0.05) levels in jejunum, as well as in a trend toward an increase in mucosal DNA and protein levels in ileum; however, this trend was not statistically significant (Figure 2). Intraperitoneal administration of ozone resulted in similar effects on mucosal DNA and protein levels compared to oral administration. The O_3_-IP rats (Group C) demonstrated a significant increase in mucosal DNA (2-fold increase, *P*<0.001) and protein (27% increase, *P*<0.05) levels in jejunum compared to control rats, as well as a trend toward an increase in mucosal DNA and protein levels in ileum; however, this trend was not statistically significant.

### Microscopic Bowel Appearance

**Figure 3.**
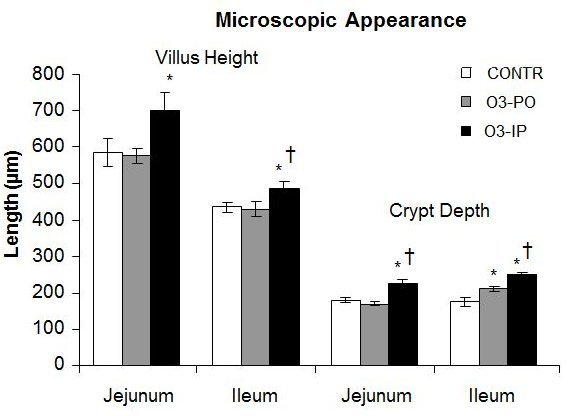
**Villus Height and Crypt Depth in Control and Ozone-Treated Rats.** Villus height and crypt depth are expressed as micro-meters. Values are provided as mean±SE. IP, intraperitoneal; O_3_, ozone; PO, per os (oral route administration). ^*^P<0.05 versus control, † *P*<0.05 O_3_-IP versus O_3_-PO.

The O_3_-PO rats (Group B) rats exhibited a significant increase in crypt depth in ileum (211±9 versus 175±13 µm, *P*<0.05) compared to control animals (Figure 3). Intraperitoneal administration of ozone (Group C) resulted in a significant increase in jejunal (702±48 versus 585±38 µm, *P*<0.05) and ileal (485±21 versus 434±13 µm, *P*<0.05) villus height, as well as in jejunal (227±9 versus 167±8 µm, *P*=0.001) and ileal (250±8 versus 175±13 µm, *P*<0.001) crypt depth compared to control animals (Group A).

### Enterocytes Proliferation and Apoptosis

**Figure 4.**
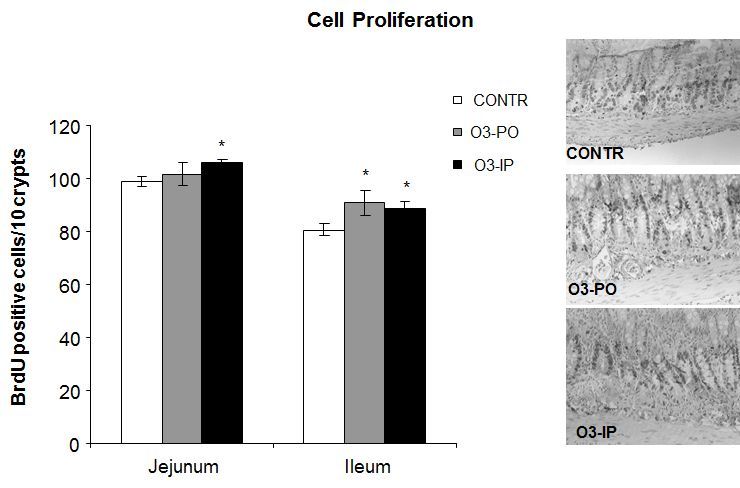
**Effect of Ozone on Cell Proliferation Rates in Jejunum and Ileum.** These representative sections demonstrate that cell proliferation is increased following ozone administration compared to control animals. Values are provided as mean±SE. IP, intraperitoneal; O_3_, ozone; PO, per os (oral route administration). ^*^P<0.05 versus control.

The O_3_-PO rats (Group B) demonstrated a trend toward an increase in the enterocyte proliferation index in ileum compared to control animals; however, this trend did not achieve statistical significance (Figure 4). Intraperitoneal ozone administration (Group C) induced a significant increase in the enterocyte proliferation index in jejunum (106±2 versus 99±2 BrdU positive cells/10 crypts, *P*=0.01) and ileum (89±2 versus 81±2 BrdU positive cells/10 crypts, *P*<0.05) compared to control animals (Group A).

**Figure 5.**
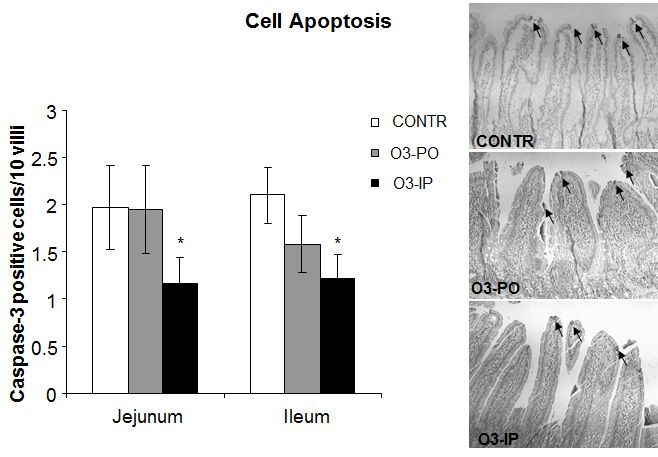
**Effect of Ozone on Enterocyte Apoptosis.** As expected, ozone-treated rats demonstrated a significant decrease in the number of apoptotic cells compared to control animals. Values are provided as mean±SE. IP, intraperitoneal; O_3_, ozone; PO, per os (oral route administration). ^*^P<0.05 versus control.

The O_3_-PO rats (Group B) showed a trend toward a decrease in the number of apoptotic cells appearing in the villi of ileum compared to control animals; however, this trend was not statistically significant (Figure 5). Treatment with IP ozone resulted in a significant decrease in the apoptotic index in ileum (1.2±0.3 versus 2.1±0.3 caspase-3 positive cells/10 villi, *P*<0.05) compared to control animals (Group A), as well as a trend toward a decrease in the apoptotic index in jejunum; however, this trend was not statistically significant.

## DISCUSSION

Ozone is one of the most controversial gases because it is useful in the stratosphere for blocking UV radiations but toxic in the troposphere during chronic air inhalation.^6^ Contrary to the old concept that ozone is always toxic, a comparative analysis of ozone levels in lungs compared to blood has fully clarified the possibility of using ozone as a therapeutic agent provided that dosages are not too high in the blood to affect antioxidant capacity.^11^ Ozone when used within the appropriate therapeutic window is absolutely safe and more effective than many other medications in various abnormalities, such as vascular diseases, infected wounds, chronic skin ulcers, initial gangrene, burns, and others. The mechanisms of ozone action have been clarified during the last two decades and include improvement in blood circulation and oxygen delivery to damaged tissues, reduction of inflammatory processes and chronic oxidative stress, enhancement in general metabolism, up-regulation of the cellular antioxidant enzyme activity, activation of immune systems, and release of growth factors.^4^,^5^ In blood, the potential energy of ozone is transferred into two fundamental messengers such as H_2_O_2_ (as a reactive oxygen species (ROS)) and aldehydic molecules of which 4-hydroxynonenal and trans-4-hydroxyhexenal are the relevant lipid oxidation products.^6^ Inflow of this small amount of H_2_O_2_ into blood cells is the indispensable stimulus to activate a series of biochemical reactions including activation of glycolysis in the erythrocytes, stimulation of neutrophil phagocytic activity, activation of tyrosine kinase in monocytes and lymphocytes, stimulation of NF-kB signaling, release of different cytokines (IFNγ and IL-8) and acute-phase proteins, and release of PDGF-αβ, TGF-β1, and IL-8 from platelets.^12^,^13^

The gastrointestinal route administration of ozone was first advocated by Aubourg^14^ who described intrarectal administration of an ozone/oxygen mixture for treating chronic colitis and fistulae. Further clinical investigations have demonstrated the positive effects of ozone therapy in several gastrointestinal disorders including methotrexate-induced intestinal injury,^7^ incompetence of intestinal anastomosis sutures,^8^ and experimental colitis.^9^ It has also been shown that O_3_ has beneficial effects in the clindamycin-associated enterocolitis model in hamsters.^15^ Recently, a preventive effect of ozone has been reported in experimental models of necrotizing enterocolitis, caustic esophageal burns, and acute necrotizing pancreatitis.^16^,^17^ The mechanisms of this positive effect of ozone therapy in the gastrointestinal tract are poorly understood.

In the current experiment, we investigated the effect of ozone on intestinal cell turnover including its effect on cell proliferation and cell death via apoptosis in rats, and we compared the parenteral (intraperitoneal) and the enteral route of ozone administration. It is obvious that enteral administration results in a significant fraction of the dose being neutralized by the intestinal environment. The residual dose of ozone will dissolve and be neutralized by a layer composed of glycocalix and muco-proteins, which covers the mucosa. Ozone will instantly and fully react with these compounds, but only a lipid oxidation products fraction will be absorbed with oxygen by the mucosa.^18^

Changes in bowel and mucosal weights, mucosal DNA and protein content, and histological appearance were measured in this study. In addition, cell proliferation and apoptosis were measured to characterize enterocyte turnover. A trophic gut effect of ozone was evidenced in this experiment by increased mucosal weight, mucosal DNA and protein, and villus height and crypt depth compared to control animals. Most significant changes were observed in the proximal intestine. Parallel increases in intestinal mucosal DNA and protein indicate that the greater mucosal mass of animals treated with ozone can be attributed to cellular hyperplasia. Because the DNA content is directly proportional to mucosal cell number, these measurements exclude such factors as edema or vascular engorgement as being responsible for differences in mucosal mass. Increased villus height may be the result of increased proliferation and accelerated migration along the villus, and is a marker for the increased absorptive surface area. Increase in crypt cells along with hypertrophy of the individual cells which we have demonstrated morphometrically is characteristic of tissues undergoing increased cell proliferation or repair. Under normal circumstances, the production of crypt cells and loss of cells at the villus tip are balanced to maintain the normal mass of gut mucosa. Measurement of cell proliferation in this study was performed using biotinylated monoclonal anti-BrdU antibody system. Ozone-treated rats demonstrated a significant increase in cell proliferation compared to control animals, and parenteral ozone exerted a significantly greater stimulating effect compared to oral route administration. It should be emphasized that up-regulation of signal transduction or release of growth factors from platelets or macrophages may be responsible for stimulating cell proliferation.

Cell loss in the small intestine is mainly regulated by programmed cell death (apoptosis). Apoptosis is a highly regulated and reproducible event that leads ultimately to cell death. Several regulatory genes affecting apoptosis have been identified and divided into pro-apoptotic genes (bax, bik, bak, bcl-xs, bad, p53, c-jun, hrk) and anti-apoptotic genes (bcl-2, bcl-x_L_, rb, mcl-1, a1, brag-1, bfl-1). Under normal circumstances, enterocyte apoptosis promotes disposal of genetically aberrant stem cells, prevents tumorigenesis, and is considered as a mechanism that counterbalances the increased enterocyte proliferation seen in order to reach a new homeostatic set during intestinal adaptation and growth. Evaluation of enterocyte apoptosis in the present study was performed using immunohistochemistry for caspase-3 and has shown a significantly decreased cell apoptosis in ozone-treated rats compared to control animals. Our observations are contrary to the recent report of pro-apoptotic effects of ozone in different cells, especially in alveolar epithelial cells.^19^ It is believed that ozone induces lipid peroxidation and that these peroxidated lipids produce oxidative stress and DNA damage.^20^ However, at low doses ozone has been shown to reduce programmed cell death through down-regulation of caspase and interleukin production.^21^

The mechanisms of the positive effects of ozone on intestinal mucosal homeostasis are poorly understood. Ozone can directly stimulate intestinal cell turnover or can act through enhancement in the release of growth factors from platelets or macrophages. The direct effect of ozone may be correlated with hydrogen peroxide production. After administration, ozone is dissolved in biological fluids such as lymph and plasma, reaches intestinal mucosa and immediately reacts with polyunsaturated fatty acids, antioxidants, reduced glutathione, and albumin, resulting in the formation of H_2_O_2_ and lipid oxidation products. An essential ROS molecule, H_2_O_2_ is able to act as an ozone messenger to elicit several biological and therapeutic effects, including regulation of signal transduction, and is a mediation of local defense and immune system responses.^22^ Indirect effects of ozone on intestinal mucosal homeostasis can be correlated with various beneficial pharmacological effects, such as edema reduction, impairment of leukocyte adhesion, enhancement of antibacterial activity, stimulation of fibro-blast proliferation and neo-vascularization, and growth factor release from platelets or macrophages. Recent evidence suggests that an ozone/oxygen mixture has modulator effects on the phagocytic activity of peritoneal and alveolar macrophages.^8^–^10^ It has been shown that O_3_ increases the activity of anti-oxidant enzymes, such as glutathione peroxidase and catalase, which protect the host from pathological conditions mediated by reactive oxygen species.^1^

In conclusion, parenteral ozone exerts intestinal trophic effects and stimulates enterocyte turnover in a rat model. Increased enterocyte proliferation and decreased cell death via apoptosis may be responsible for this positive effect.

## Abbreviations:

AI: apoptotic index;
IP: intraperitoneally;
ROS: reactive oxygen species.
